# Blood Flow Assessment of Arteriovenous Malformations Using Intraoperative Indocyanine Green Videoangiography

**DOI:** 10.1155/2019/7292304

**Published:** 2019-03-17

**Authors:** Naoki Kato, Vincent Prinz, Julius Dengler, Peter Vajkoczy

**Affiliations:** Department of Neurosurgery, Charité Universitätsmedizin Berlin, Germany

## Abstract

Intraoperative indocyanine green (ICG) videoangiography is widely used in patients undergoing neurosurgery. FLOW800 is a recently developed analytical tool for ICG videoangiography to assess semi-quantitative flow dynamics; however, its efficacy is unknown. In this study, we evaluated its functionality in the assessment of flow dynamics of arteriovenous malformation (AVM) through ICG videoangiography under clinical settings. ICG videoangiography was performed in the exposed AVM in eight patients undergoing surgery. FLOW800 analysis was applied directly, and gray-scale and color-coded maps of the surgical field were obtained. After surgery, a region of interest was placed on the individual vessels to obtain time-intensity curves. Parameters of flow dynamics, including the maximum intensity, transit time, and cerebral blood flow index, were calculated using the curves. The color-coded maps provided high-resolution images; however, reconstruction of colored images was restricted by the depth, approach angle, and brain swelling. Semi-quantitative parameters were similar among the feeders, niduses, and drainers. However, a higher cerebral blood flow index was observed in the feeders of large AVM (>3 cm) than in those of small AVM (P < 0.05). Similarly, the cerebral blood flow index values were positively correlated with the nidus volume (P < 0.01). FLOW800 enabled visualization of the AVM structure and safer resection, except in case of deep-seated AVM. Moreover, semi-quantitative values in the individual vessels through using ICG intensity diagram showed different patterns according to size of the AVM. ICG videoangiography showed good performance in evaluating flow dynamics of the AVM in patients undergoing surgery.

## 1. Introduction

Patients with arteriovenous malformation (AVM) are usually treated by means of direct surgery, endovascular embolization, or stereotactic radiotherapy, of which direct surgery is most effective to improve the patients' long-term prognosis. Individualized approach using intraoperative neurophysiological monitoring, neuronavigation, or intraoperative digital subtraction angiography (DSA) is recommended for adequate treatment of patients with AVM [1–4]. Recently, intraoperative indocyanine green (ICG) videoangiography has become an indispensable tool for use in patients undergoing neurovascular surgery [5–8].

In addition, FLOW800 (Zeiss OPMI Pentero, Carl Zeiss Meditec, Oberkochern, Germany), a newly developed analytical tool in ICG videoangiography, has recently become available for clinical use to obtain qualitative visualization of the blood flow and vessel strength [9–11]. This function provides useful information about the operative fields' structure with detailed color-coded images, as well as semi-quantitative data through time-intensity curves for analysis of the blood flow parameters of target areas [12, 13]. Kamp et al. reported mean values of the parameters in patients undergoing different surgical procedures, including treatment of aneurysm, brain ischemia, and AVM, using the time-intensity diagram of ICG; with regard to each parameter, the results indicated significant differences between the normal brain cortex and the less perfused areas [14]. The unique features of AVMs, i.e. their hypervascularization and large caliber vessels, pose a different challenge towards the assessment of the flow dynamics than aneurysm cases. Since adequate flow reduction of the nidus is effective in avoiding bleeding and can be the aid of resection of the AVM, detection of high flow feeders during an early stage is important. We assumed that the color-coded map or diagram of each vessel has a potential of detecting feeders or confirming flow reduction. Based on these points, our study aimed to validate the function of FLOW800 in treatment procedures of the AVM under clinical setting and comparatively evaluate semi-quantitative parameters in the individual vessels of AVMs with specific characteristics.

## 2. Materials and Methods

A total of consecutive seventeen patients who received surgical resection of intracranial AVM were retrospectively detected in this study. As the data obtained from routine examination was analyzed, nine patients who underwent microsurgery with conventional microscope were excluded. Of the seventeen patients, the remaining eight patients who were treated using intraoperative ICG videoangiography with FLOW800 function were analyzed. Each AVM was classified per Spetzler and Martin grading system [15]. The diameter of the nidus was obtained under condition of DSA imaging system equipped with size-measurement devices or through multiplanar reconstruction using computed tomography (CT) imaging or magnetic resonance imaging (MRI) with contrast enhancement. The nidus volume was calculated using the formula of cubage as follows [16]:

(1) $$\begin{array}{c} Nidus\;\;volume=\frac{\pi }{6}\cdot a\cdot b\cdot c \end{array}$$

a, b, c are maximum diameters (cm) of three perpendicular dimensions; *π* is Pi.

**Figure 1:**
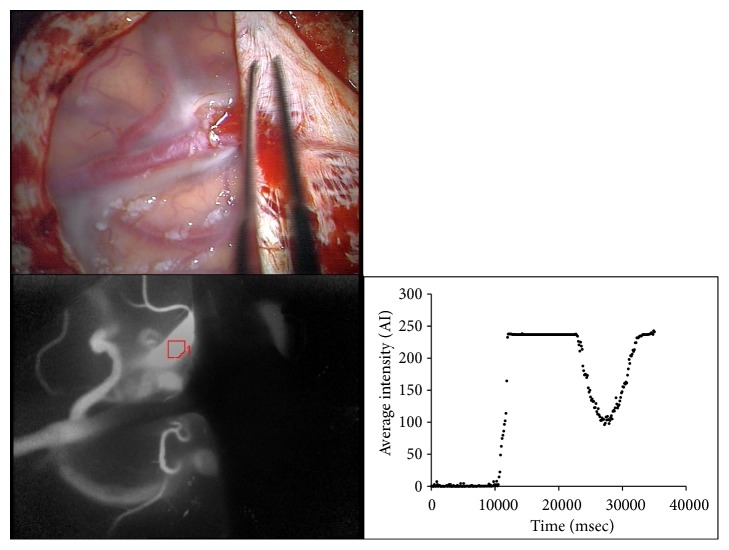
Example of ROI placement in an individual vessel of the AVM. Diagram showing the time-intensity curve of ICG in the individual vessel with ROI placement.

After surgical exposure of the AVM, ICG videoangiography was performed in the usual manner to obtain a maximum intensity map with gray-scale and color-coded images indicating the delay time of the exposed operative field. In each angiography procedure, ICG 25 mg dissolved in 5-ml water was administered. In addition, to obtain diagrams for semi-quantitative assessment, ROIs were placed on the individual vessels including the feeders, niduses, and drainers in the exposed area (Figure 1); in case of multiple incorporated feeders or drainers in the AVM, all were included for analysis; in case of the adequately exposed nidus, an ROI was placed on one spot of the center of the nidus as much as possible; and other vessels that were not within the exposed area were excluded from the ROI placement. The size and the form of the ROI were adjusted to the vessel structure automatically by the FLOW800 software function. Next, we obtained the following parameters from the time-intensity curves of the ROIs comprising individual target vessels. Maximum intensity was defined as the maximum value of ICG intensity curve in arbitrary intensity units [AI]. Delay time was defined as the time interval between the appearance and half-maximum intensity of ICG fluorescence [s]. Cerebral blood flow index (CBFi) was obtained from the maximum intensity divided by the time interval between 10% and 90% of the maximum fluorescence signal [AI/sec] [17–19]. All values were calculated manually using an Excel tool (Microsoft Excel, Microsoft) in order to exclude artefacts.

All patients underwent postoperative DSA to confirm complete resection of the AVM. The patients' short-term prognosis on the day of discharge was evaluated using the modified Rankin Scale (mRS).

### 2.1. Statistical Analysis

One-factor analysis of variance (ANOVA) was used for the comparison of three parameters according to vessel type. T-test was performed for comparisons of the nidus diameter and CBFi, after confirming normal distribution pattern using F-test. Correlations between the nidus volume and CBFi were evaluated using Spearman's rank correlation test. Statistical significance was defined as P-value less than 0.05. All statistical analyses were performed with Statview (version 5.0, SAS institute, North Carolina, USA).

## 3. Results

The patients' characteristics, size and location of AVM, number of ROIs, clinical presentation, and short-term outcome are shown in Table 1. All patients were anesthetized intra-operatively using remifentanyl combined with propofol or midazolam. The range of systolic blood pressure was 104–122 mmHg (mean, 110 mmHg) and heart rate was 46–79 bpm (mean, 55.4 bpm), during the time-period of ICG injection and fluorescence recording. In all cases, the recordings were successfully performed without complications. In addition, complete removal of the AVM was confirmed using postoperative DSA. The patients' mean hospital stay was 11.6 days (range, 4–32).

### 3.1. Map Reconstruction

In all cases, immediate reconstruction of the color-coded map was achieved intraoperatively. The delay map showed the surface structure of the AVM with information regarding direction and velocity of blood flow in the exposed vessels. Due to feature of the visualization method, the border between the normal brain cortex and nidus was more evident, enabling accurate determination of the incision point and dissection plane; additionally, the main feeders were clearly visualized and easily occluded. Nevertheless, in some cases, the AVM could not be visualized adequately due to depth of the nidus, angle of surgical approach, or post-hemorrhage swelling of the brain.

### 3.2. Assessment of Semi-Quantitative Parameters

**Figure 2:**
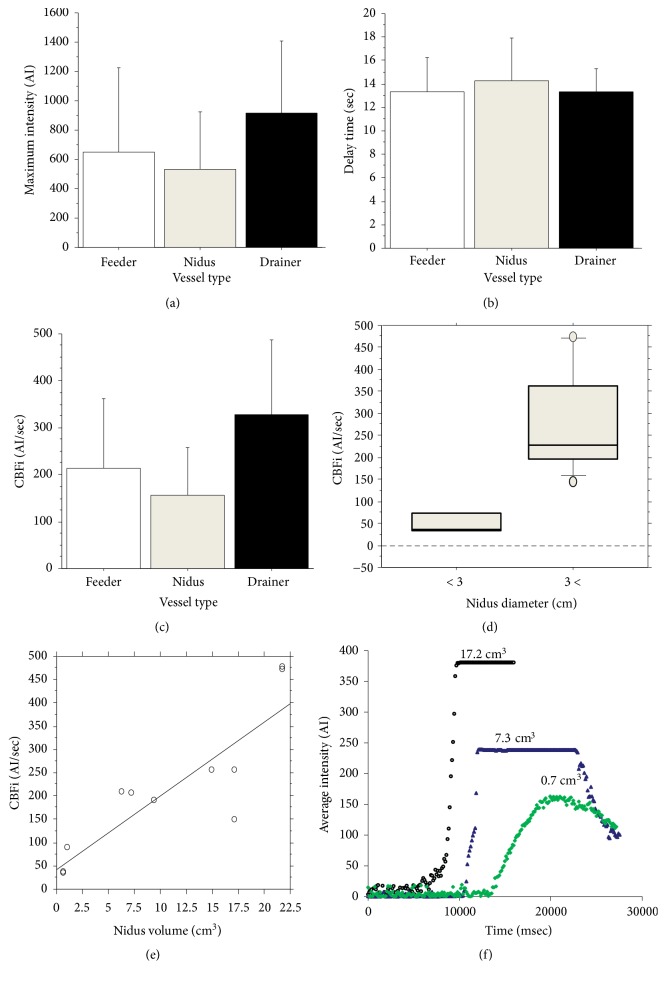
Comparison of parameters according to vessel types. Maximum intensity (a), delay time (b), and CBFi (c). Values showed absence of significant differences among the vessel types. The CBFi value of the feeders was significantly higher in the niduses of > 3-cm diameter (d). In addition, the CBFi of the feeders was correlated with the cubage of the nidus (e). Representative time-intensity diagrams presenting three different patterns according to the nidus volume (f). CBFi: cerebral blood flow index.

In semi-quantitative calculation of flow dynamics through time-intensity diagrams, a total of 28 ROIs were placed on the individual vessels of the AVMs. In total, 11 diagrams of the feeders, seven of the niduses, and 10 of the draining veins were obtained; the three parameters according to vessel types, i.e., the feeder, nidus, or draining vein, were calculated. All data are presented as mean ± standard deviation (SD). The mean maximum intensity was 652.8 ± 571.9 AI in the feeders, 534.3 ± 392.7 AI in the niduses, and 915.6 ± 495.1 AI in the draining veins; the mean delay time was 13.3 ± 2.89 sec in the feeders, 14.2 ± 3.64 sec in the niduses, and 13.3 ± 2.03 sec in the draining veins; and the mean CBFi was 212.9 ± 149.2 AI/sec in the feeders, 156.7 ± 102.2 AI/sec in the niduses, and 326.9 ± 161.2 AI/sec in the draining veins (Table 2). The values of the three parameters did not exhibit significant differences based on the vessel type (Figures 2(a), 2(b), and 2(c)).

Comparison of the CBFi value in all the feeders according to the nidus diameter indicated the presence of higher CBFi values in the large niduses (>3 cm) versus the small niduses (P < 0.05) (Figure 2(d)), with mean value of 50.9 ± 30.4 AI/sec in the small niduses, and 273.7 ± 126.8 AI/sec in the large niduses (Table 3). In addition, the CBFi value showed a positive correlation with the nidus volume (P < 0.01) (Figure 2(e)). These findings suggested that ICG flows more rapidly into the large niduses than the small niduses (Figure 2(f)).

### 3.3. Representative Cases

#### 3.3.1. Case 3

**Figure 3:**
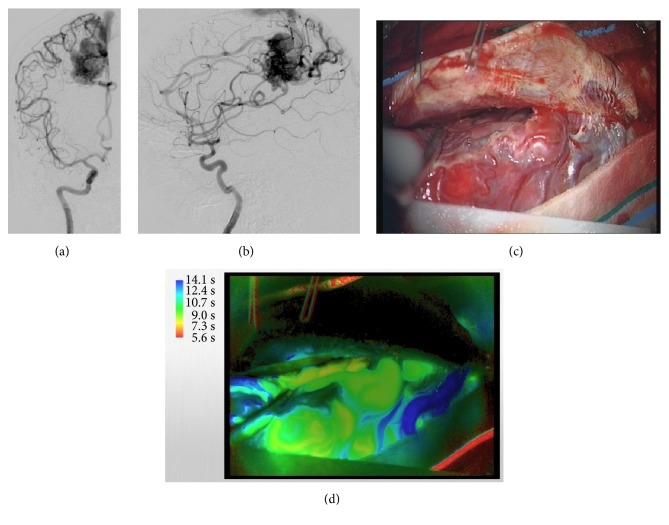
Case 3. DSA revealed the presence of AVM in the right parasagittal postcentral region (a, b). Intraoperative photograph at after dural opening demonstrating restricted exposure of the AVM (c). Delay map using FLOW800 showed poor performance in demonstrating the accurate structure of the entire AVM (d).

A 51-year-old woman who experienced massive intraventricular hemorrhage due to presence of a post-central parasagittal AVM, Spetzler & Martin grade 3, underwent surgery (Figures 3(a) and 3(b)). In this case, we performed right frontoparietal craniotomy and approached the AVM via right interhemispheric route (Figure 3(c)). However, through the delay map, visualization of the precise structure of the AVM was not possible due to depth of the nidus and angle of the surgical approach (Figure 3(d)); moreover, due to swelling of the brain, exposure of the AVM was unstable.

#### 3.3.2. Case 5

**Figure 4:**
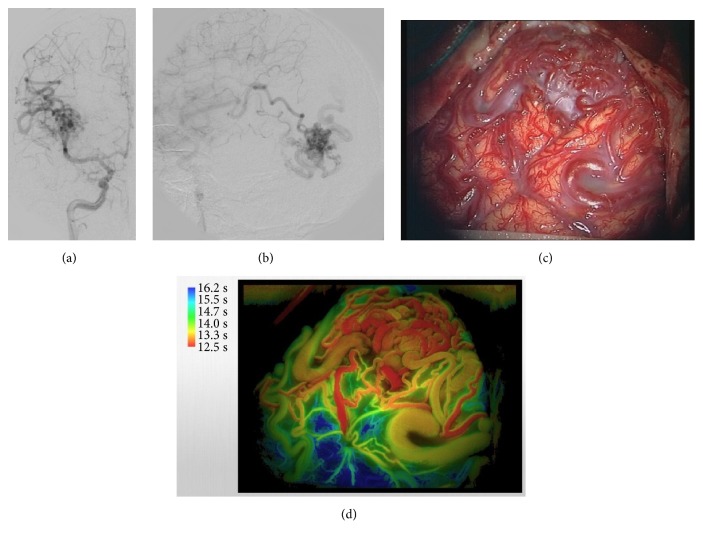
Case 5. Preoperative DSA showed an AVM in the right occipital lobe (a, b). Intraoperative photograph at after dural opening demonstrating surface appearance of the AVM (c). Delay map showing the feeders and accurate location of the nidus (d).

A 33-year-old man with an unruptured right-occipital AVM underwent surgical treatment (Figures 4(a) and 4(b)). Intraprocedural delay mapping provided a detailed color-coded image after opening of the dura (Figures 4(c) and 4(d)); and the location of the nidus and main feeders was visualized. Based on the findings, we occluded the feeders and achieved complete removal of the AVM with accurate dissection plane between the nidus and normal brain tissue.

## 4. Discussion

Intraoperative ICG videoangiography is widely used and has become an indispensable imaging modality for use in patients undergoing neurovascular surgery [6–8]. This method can be used to detect the residual nidus or angiographically-occult shunting in surgical treatment of the AVM [4, 20]. Intraoperative DSA is an effective tool for visualization of the lesion; however, it is not always available in every institute and associated with potential risks such as catheter migration, thrombosis, or radiation exposure [21, 22]. Moreover, intraoperative ICG videoangiography can be performed noninvasively resulting in less delay during surgery, which is advantageous over intraoperative DSA [11, 23]. In recent years, FLOW800, the newly developed analytical tool for use in ICG videoangiography, has become available for clinical use [9–11]. Injected ICG passes rapidly through the veins, capillaries, and arteries. Based on the arrival time of ICG, it is possible to distinguish between high-velocity blood flow and low-velocity blood flow. Color-coded maps are generated indicating the time interval between the half peak and appearance of ICG in the operative field. Images may be acquired within 2-min period after initiating the recording. Thus, the flow velocity in the exposed area can be measured intraoperatively. In areas involving pathological process, such as the ischemic core or arteriovenous shunts, the color-coded map shows changes according to the blood flow, which enables detection of the feeders of the AVM or the direction of blood flow within the vessels [9, 14]. In addition, FLOW800 has functionality to calculate semi-quantitative parameters from the time-intensity curves of ICG in the ROI [9, 14].

Woitzik et al. first introduced the intensity curve analysis method [13]; the intensity curves displayed different patterns based on differential perfusion levels of the cortex such as that of the ischemic core or penumbra. Kamp et al. reported the average values of parameters related to blood flow in the aneurysm, Moyamoya disease, and AVM using FLOW800, indicating significantly lower CBFi in the ischemic brain cortical areas than in other physiological locations [14].

In the present study, we calculated similar parameters in the individual vessels and compared characteristics between the vessels or niduses. All semi-quantitative values obtained through intensity curves were similar among the feeders, niduses, and drainers, which may be due to presence of abnormally high flow in the draining veins from direct supply through the feeders via the nidus, a common occurrence in the AVM.

In contrast, the CBFi values in the feeders correlated positively with the diameter and volume of the nidus. Similar results regarding the relationship between the blood flow and size of the nidus have been reported by previous study using indirect methods. A study using echocardiogram-triggered phase contrast MRI showed that the cerebral blood flow (CBF) was markedly higher in the ipsilateral versus contralateral internal carotid artery and correlated with the AVM volume [16]. A study using transcranial Doppler ultrasonography likewise indicated the presence of higher blood flow velocity in the ipsilateral MCA that returned to normal level after ablation of the AVM through radiosurgery [24]. In each context, the indirect CBF measurement showed positive correlations between the nidus volume and CBF of the incorporated arteries in the AVM. In our study, direct measurement using intraoperative ICG videoangiography demonstrated a similar trend and enabled evaluation of blood flow in the individual vessels of the AVM. If high flow feeders are detected with the same manner, immediate feeder trapping of the AVM can be easily achieved for following safe resection of the nidus. Besides, we assume that the flow analysis using FLOW800 has a potential to confirm sufficient flow reduction of the nidus before radical dissection between the AVM and the normal brain tissue.

Color-coded delay maps revealed the surface structure of the AVM as well as the main feeders, facilitating accurate sulcotomy prior to resection of the nidus. However, in the event of deep-seated location of the AVM within the brain tissue, or poor exposure, acquired images would be inadequate due to the depth and angle of the operative field [10, 11]. A limitation of our study included restricted exposure of the AVMs or heterogenous structure of the nidus, which may affect the interpretation of semi-quantitative analysis due to selection bias of the vessels with ROIs placement. Accurate evaluation of the semi-quantitative values of blood flow is compromised in the feeders, niduses, or drainers that are not adequately exposed. This effect must be considered in the interpretation of results. In deep-seated AVM structures, intraoperative DSA or endoscopy with mounted ICG videoangiography system may be a helpful alternative [25]. In the present study, anesthesiologists were not provided with specification of the exact method of ICG injection, which may result in technical bias. Moreover, some diagrams showed a plateau line at the maximum intensity as the presented figures due to the halation of the ICG illumination that was associated with gain value of the sensor. This factor can be the risk affecting the mean value of each semiquantitative parameters as artifact. Nevertheless, the benefits of easy handling and daily use of the technique is the main advantage of intraoperative ICG videoangiography compared to intraoperative DSA [8, 10].

## 5. Conclusions

In conclusion, FLOW800 provided helpful information to surgeons aiming to develop optimal surgical strategy in treatment of patients with AVMs. Moreover, our data suggested that semi-quantitative assessment of ICG videoangiography may be a useful tool for evaluating flow dynamics in patients with neurovascular disease.

## Figures and Tables

**Table 1:** Patients characteristics.

Case ID	Age	Location	SM grade	Nidus	Leading	Postoperative	No. of ROI			Residual
	(years), Sex			volume (cm^3^)	symptom	mRS	F	N	D	Nidus
1	22, M	Left frontal	1	7.3	Epilepsy	0	1	1	2	-
2	29, M	Left postcentral	2	1.1	Numbness	0	1	1	N/A	-
3	51, W	Right postcentral	4	17.2	IVH	6	2	1	1	-
4	28, W	Right cerebellar	2	0.7	ICH	4	2	1	N/A	-
5	33, M	Right occipital	3	15.0	Migraine	0	1	1	2	-
6	48, W	Left precentral	2	6.4	None	4	1	1	2	-
7	26, M	Right temporo- occipital	3	21.8	IVH	0	2	N/A	2	-
8	72, W	Left temporal	3	9.5	SAH	3	1	1	1	-

SM, Spetzler & Martin; IVH, intraventricular hemorrhage; ICH, intracerebral hemorrhage; F, feeder; N, nidus; D, draining vein; N/A, not available.

**Table 2:** Mean value of each parameters regarding vessel type.

	Feeder	Nidus	Draining vein
Mean maximum intensity ± SD (AI)	652.8 ± 571.9	534.3 ± 392.7	915.6 ± 495
Mean delay time ± SD (sec)	13.3 ± 2.89	14.2 ± 3.64	13.3 ± 2.03
Mean CBFi ± SD (AI/sec)	212.9 ± 149.2	156.7 ± 102.2	326.9 ± 161.2

**Table 3:** Comparison of CBFi according to the size of nidus.

	Small nidus	Larger nidus
	(< 3cm)	(> 3cm)
Mean CBFi ± SD (AI/sec)	50.9 ± 30.4	273.7 ± 126.8

## Data Availability

The data used to support the findings of this study are included within the article.
